# Retrospective analysis of transarterial chemoembolization and sorafenib in Chinese patients with unresectable and recurrent hepatocellular carcinoma

**DOI:** 10.18632/oncotarget.11514

**Published:** 2016-08-23

**Authors:** Xuying Wan, Xiaofeng Zhai, Zhenlin Yan, Pinghua Yang, Jun Li, Dong Wu, Kui Wang, Yong Xia, Feng Shen

**Affiliations:** ^1^ Department of Combined Traditional Chinese and Western Medicine, The Eastern Hepatobiliary Surgery Hospital, Second Military Medical University, Shanghai, China; ^2^ Department of Traditional Chinese Medicine, The Changhai Hospital, Second Military Medical University, Shanghai, China; ^3^ Department of Hepatic Surgery, The Eastern Hepatobiliary Surgery Hospital, Second Military Medical University, Shanghai, China

**Keywords:** hepatocellular carcinoma, sorafenib, survival, transarterial chemoembolization

## Abstract

We explored the hypothesis that sorafenib may improve the effect of transarterial chemoembolization (TACE) in patients with recurrent hepatocellular carcinoma (HCC) and that longer sorafenib duration was associated with additional survival benefits. In this retrospective, nested case-controlled study, 1126 cases of unresectable HCC were collected. Patients with unresectable disease treated with TACE+sorafenib (n=245) and TACE alone (n=245) and those with recurrence after surgery treated with TACE+sorafenib (n=127) and TACE alone (n=127) were identified and matched according to sex, age, and lesion size and number. The clinicopathological factors associated with survival were examined by univariate and multivariate analyses. The mean duration of sorafenib treatment was 10.8±10.51 months. Sorafenib significantly increased the median survival time as compared to TACE alone (unresectable HCC: 20.23 vs. 13.97 months, respectively; *p*=0.013 and recurrent HCC: 30.7 and 18.22 months, respectively; *p*=0.003). The survival of patients with unresectable HCC was associated with the presence of portal vein tumor thrombus (HR=1.47, *p*=0.004) and treatment method (TACE+sorafenib combination therapy; HR=0.72, *p*=0.003). For patients with recurrent HCC, the presence of extrahepatic metastasis (HR=1.71, *p*=0.012) and treatment method (TACE+sorafenib therapy; HR=0.60, *p*=0.002) also was associated with survival. For patients treated with TACE+sorafenib, multivariate analysis showed decreased hazard of death with longer duration of sorafenib treatment (HR=0.9, *p*<0.001). Thus, sorafenib plus TACE may provide survival benefits, which may be related with sorafenib treatment duration, particularly for patients with HCC recurrence. Further clinical studies are required to confirm these results and identify which patients are most likely to benefit from this therapeutic strategy.

## INTRODUCTION

Liver cancer is the fifth most frequently diagnosed cancer worldwide and the second leading cause of cancer-related death [1]. A majority of patients with liver cancer are diagnosed with hepatocellular carcinoma (HCC). For many HCC patients, hapatectomy and orthotopic liver transplantation are unsuitable due to the advanced stage at initial diagnosis [2,3]. For patients with unresectable HCC, transarterial chemoembolization (TACE) is the standard therapy.

In addition to TACE, sorafenib, a tyrosine kinase inhibitor [4] that suppresses HCC cell proliferation and angiogenesis, is used for patients with advanced HCC [5]. Sorafenib suppression of tumor growth and metastasis by STAT3 inhibition was also shown in a rat HCC model [6]. In addition, the efficacy of sorafenib as a first-line therapy for advanced HCC was reported in the SHARP (Sorafenib HCC Assessment Randomized Protocol) trial [7–10]. Specifically, sorafenib may provide survival benefits for patients with advanced HCC [11] as well as recurrent HCC following liver transplantation [12]. With the exception of HCC patients with Child-Pugh class B liver function [13, 14], sorafenib has a generally favorable safety profile [15–18]. Its wide application, however, is limited by its induction of resistance in some patients as well as its high cost [19], and availability, in particularly for Chinese patients.

The European Association for the Study of the Liver (EASL) guideline for the treatment of HCC does not recommend initiating sorafenib when the tumor responds well (CR+PR) to TACE [20]. In a phase III study conducted by Kudo et al. [21], addition of sorafenib did not improve time to progression in patients who responded to TACE; however, the median duration of sorafenib therapy was 17.1 weeks, and more than half of the patients started sorafenib >9 weeks after TACE. Subsequent subgroup analysis that included 458 patients revealed that Korean patients who were treated with sorafenib for a much longer period than the Japanese patients (31 vs. 16 weeks, respectively) revealed better outcomes (i.e., time to progression) in those treated with the combination therapy [21].

Continual use of sorafenib was effective in renal tumor patients with disease progression [22], and anecdotal evidence from our institution suggests that sorafenib efficacy increases with its duration of use. However, studies assessing the effects of long-term sorafenib therapy for HCC patients have reported conflicting results [21, 23]. Thus, this retrospective study aimed to test the hypothesis that the addition of sorafenib to TACE, has the potential to improve the efficacy of TACE in patients with recurrent and unresectable HCC and that early initiation of sorafenib can provide additional survival benefits for patients with advanced disease. Thus, we focused on patients with recurrent diseases because they usually had a relatively better baseline compared with the studies from other centers in China [24]. We also identified the clinicopathological factors associated with survival in these patients.

## RESULTS

### Demographic distribution of the study participants

A total of 490 patients with advanced HCC were enrolled in this study, including 245 patients (218 males and 27 females) treated with TACE+sorafenib and 245 patients (218 males and 27 females) treated with TACE (Table 1). Both groups had 115 subjects aged <50 y. Significant differences in α-fetoprotein (AFP) levels, the presence of ascites and portal vein tumor thrombus (PVTT), and Eastern Cooperative Oncology Group (ECOG) status were detected between the treatment groups (all *p*≤0.036). Specifically the proportion of subjects with AFP levels ≥400 μg/L was higher in TACE+sorafenib group than the TACE group (*p*<0.001). No significant differences in tumor size and number, the presence of hepatic cirrhosis or extrahepatic metastasis or Child-Pugh scores were found between the groups (Table 1).

**Table 1:** Demographic distribution of the study participants

	TACE+sorafenib (N=245)	TACE (N=245)	*p*-value
Age (y)			1.000
<50	115 (46.9%)	115 (46.9%)	
≥50	130 (53.1%)	130 (53.1%)	
Gender			1.000
Male	218 (89%)	218 (89%)	
Female	27 (11%)	27 (11%)	
AFP (μg/L)			<0.001
<400	132 (53.9%)	170 (69.4%)	
≥400	113 (46.1%)	75 (30.6%)	
Tumor size (cm)			1.000
<5	153 (62.4%)	153 (62.4%)	
≥5	92 (37.6%)	92 (37.6%)	
Tumor number			1.000
Single	107 (43.7%)	107 (43.7%)	
Multiple	138 (56.3%)	138 (56.3%)	
Ascites	14 (5.7%)	5 (2%)	0.036
Hepatic cirrhosis	114 (46.5%)	106 (43.4%)	0.493
PVTT	70 (28.6%)	27 (11.1%)	<0.001
Extrahepatic metastasis	43 (17.6%)	36 (14.7%)	0.390
Child-Pugh			0.488
A	213 (86.9%)	218 (89%)	
B	32 (13.1%)	27 (11%)	
ECOG status			<0.001
0/1	223 (91.0%)	162 (66.1%)	
2	22 (9.0%)	83 (33.9%)	

TACE, transarterial chemoembolization; AFP, α-fetoprotein; PVTT, portal vein tumor thrombus; ECOG, Eastern Cooperative Oncology Group

Of the patients in the TACE+sorafenib group, 86 subjects had hand and foot skin reaction, 76 subjects had diarrhea, 116 subjects experienced hair loss, 58 subjects had fatigue, 89 subjects developed rash, 8 subjects had hypertension, 31 subjects had anorexia, 7 subjects experienced nausea, and 30 subjects developed other adverse events (e.g., pain, hand pain, sore foot, etc).

### Univariate and multivariate analyses to identify factors associated with survival

As shown in Table 2, univariate analysis revealed that PVTT was associated with increased hazard of death (HR=1.37, *p*=0.016). In addition, subjects treated with TACE+sorafenib had a significantly lower hazard of death compared with those treated with TACE alone (HR=0.76, *p*=0.013; Table 2). After adjusting for PVTT, multivariate analysis revealed that subjects treated in TACE+sorafenib continued to have a significantly lower hazard of death compared with those treated with TACE alone (HR=0.72, *p*=0.003; Table 2).

**Table 2:** Factors associated with survival in patients with unresectable HCC

	Univariate		Multivariate	
	HR (95% CI)	*p*-value	HR (95% CI)	*p*-value
PVTT				
No	Ref		Ref	
Yes	1.37 (1.06 - 1.77)	0.016	1.47 (1.14 - 1.91)	0.004
Group				
TACE+sorafenib	0.76 (0.61 - 0.94)	0.013	0.72 (0.57 - 0.89)	0.003
TACE	Ref		Ref	

TACE, transarterial chemoembolization; PVTT, portal vein tumor thrombus

No significant affect in survival were found in age, gender, α-fetoprotein, Child-Pugh, Tumor number, Tumor size, Extrahepatic metastasis, and Eastern Cooperative Oncology Group status.

**Figure 1:**
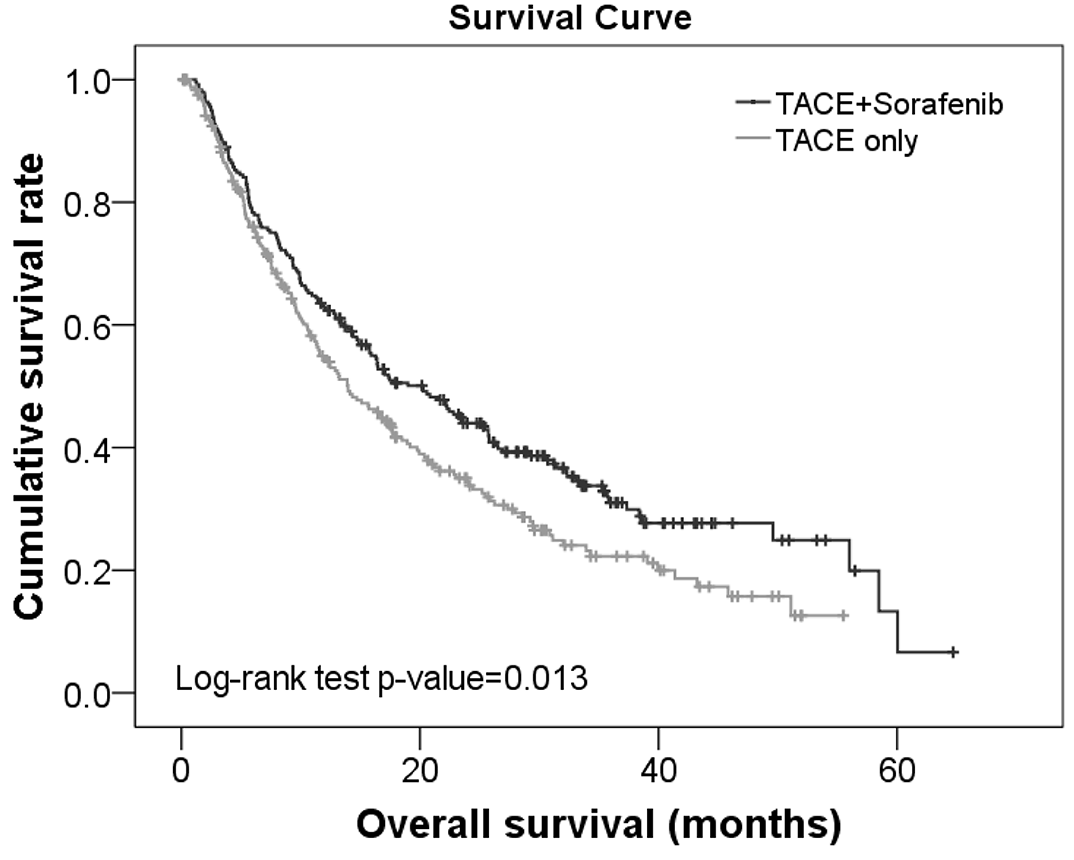
Survival curves for patients with unresectable HCC treated with TACE+sorafenib or TACE alone.

Analysis of the survival curves revealed that the survival rates for patients treated with TACE+sorafenib was significantly higher compared with those receiving TACE alone (*p*=0.013; Figure 1). Specifically, the median survival time was 20.23 and 13.97 months in subjects treated with TACE+sorafenib and TACE alone, respectively. Furthermore, the 1-, 2-, and 3-year survival rates for patients in the TACE+sorafenib group were 62.73%, 43.96%, and 31.03%, respectively. In the TACE group, the 1-, 2-, and 3-year survival rates were 54.93%, 34.40%, and 22.27%, respectively.

Kaplan Meier survival analysis also revealed that patients treated with TACE+sorafenib and without PVTT had significantly higher survival rates than those treated with TACE+sorafenib but with PVTT (median survival time: 23.33 vs. 12.73 months, respectively; *p*≤0.044; Supplementary Figure S1). The survival rates of these patients were also significantly higher than those treated TACE without and with PVTT (median survival time: 23.33 vs. 13.90 and 15.10 months, respectively; *p*≤0.044; Supplementary Figure S1).

### Demographic characteristics of subjects with recurrence

Of the 354 subjects with recurrence after surgery, there were 116 males and 11 females with 69 subjects aged <50 y in each treatment group. As shown in Table 3, AFP and PVTT were significantly different between the treatment groups (both *p*<0.001). The proportion of subjects with AFP levels ≥400 μg/L was greater in the TACE+sorafenib group. No significant differences in tumor size and number as well as the presence of ascites, hepatic cirrhosis, extrahepatic metastasis, Child-Pugh, and ECOG score were found (Table 3).

**Table 3:** Demographic distribution of the patients with disease recurrence

	TACE+sorafenib (N=127)	TACE only (N=127)	*p*-value
Age (y)			1
<50	69 (54.3%)	69 (54.3%)	
≥50	58 (45.7%)	58 (45.7%)	
Gender			1
Male	116 (91.3%)	116 (91.3%)	
Female	11 (8.7%)	11 (8.7%)	
AFP (μg/L)			<0.001
<400	69 (54.3%)	98 (77.2%)	
≥400	58 (45.7%)	29 (22.8%)	
Tumor size (cm)			1
<5	99 (78%)	99 (78%)	
≥5	28 (22%)	28 (22%)	
Tumor number			1
Single	54 (42.5%)	54 (42.5%)	
Multiple	73 (57.5%)	73 (57.5%)	
Ascites	8 (6.3%)	2 (1.6%)	0.053
Hepatic Cirrhosis	72 (56.7%)	57 (45.2%)	0.068
PVTT	30 (23.6%)	4 (3.1%)	<0.001
Extrahepatic metastasis	23 (18.1%)	15 (11.8%)	0.159
Child-Pugh			0.058
A	110 (86.6%)	119 (93.7%)	
B	17 (13.4%)	8 (6.3%)	
ECOG status			0.065
0/1	115 (90.6%)	105 (82.7%)	
2	12 (9.4%)	22 (17.3%)	

TACE, transarterial chemoembolization; AFP, α-fetoprotein; PVTT, portal vein tumor thrombus; ECOG, Eastern Cooperative Oncology Group

### Factors associated with survival in patients with disease recurrence

Univariate analysis revealed that extrahepatic metastasis and TACE+sorafenib treatment were associated with survival in patients with disease recurrence. Specifically, the presence of extrahepatic metastasis was negatively associated with survival (HR=1.6, *p*=0.028; Table 4). In addition, subjects treated with TACE+sorafenib had a significantly lower hazard of death compared with those treated with TACE alone (HR=0.62, *p*=0.004; Table 4). After adjusting for the presence of extrahepatic metastasis, subjects treated with TACE+sorafenib had significantly lower hazard of death compared with those treated in TACE only (HR=0.6, *p*=0.002; Table 4).

**Table 4:** Factors associated with survival in patients with disease recurrence

	Univariate		Multivariate	
	HR (95% CI)	p-value	HR (95% CI)	p-value
Extrahepatic metastasis				
No	Ref		Ref	
Yes	1.6 (1.05 - 2.42)	0.028	1.71 (1.12 - 2.6)	0.012
Group				
TACE+sorafenib	0.62 (0.45 - 0.86)	0.004	0.6 (0.43 - 0.83)	0.002
TACE	Ref		Ref	

TACE, transarterial chemoembolization

No significant affect in survival were found in age, gender, α-fetoprotein, Child-Pugh, tumor number, tumor size, portal vein tumor thrombus, extrahepatic metastasis, and Eastern Cooperative Oncology Group status.

**Figure 2:**
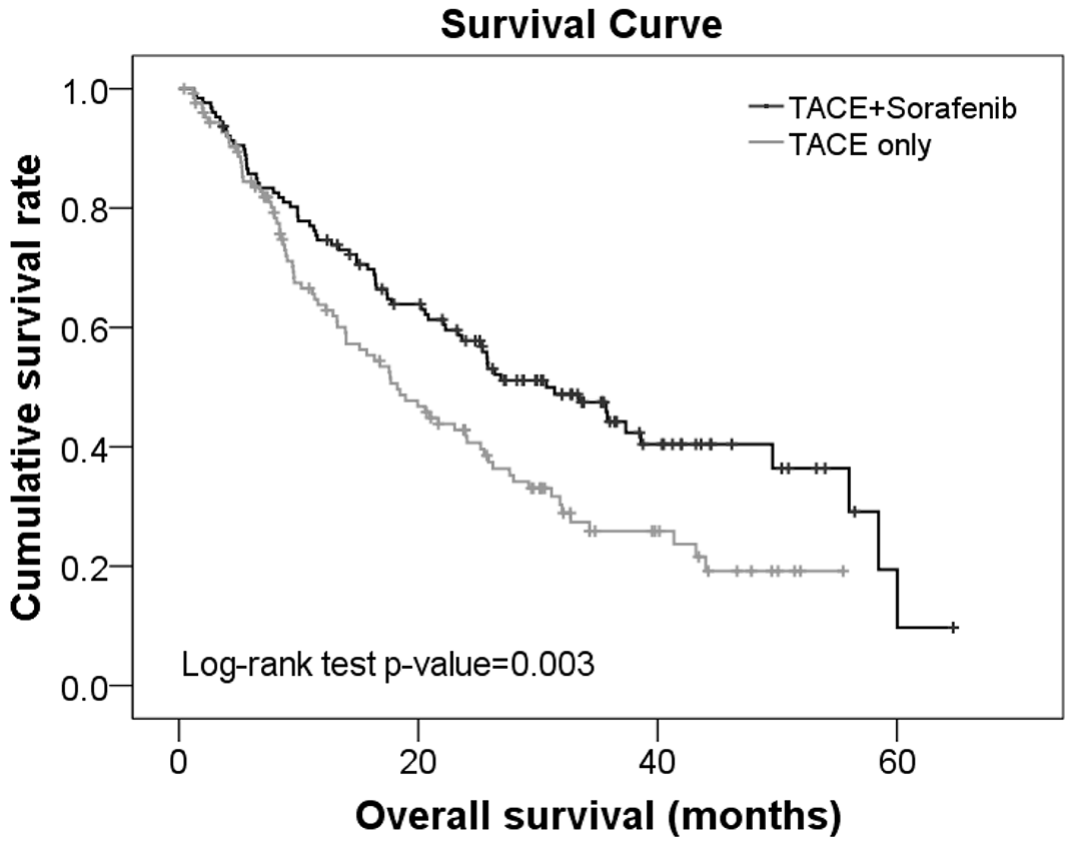
Survival curves for advanced HCC patients treated with TACE+sorafenib or TACE alone with disease recurrence.

Kaplan Meier survival curves revealed that the survival rate was significantly higher in the TACE+sorafenib group compared with TACE alone group (*p*=0.003; Figure 2). The median survival time was 30.7 and 18.22 months in subjects treated with TACE+sorafenib and TACE, respectively. Furthermore, the 1-, 2-, and 3- survival rates for patients treated with TACE+sorafenib were 74.64%, 57.78%, and 44.21%, respectively; they were 63.79%, 41.76%, and 25.87%, respectively for those treated with TACE alone.

Of patients treated with TACE+sorafenib, those without extrahepatic metastasis had significantly higher survival rates than those with extrahepatic metastasis (median survival time: 35.8 vs. 22.13 months, respectively, *p*≤0.019; Supplementary Figure S2). The rates were also higher than patients treated with TACE both without and with extrahepatic metastasis (median survival time: 35.8 vs. 18.93 and 11.17 months, respectively; *p*≤0.019; Supplementary Figure S2).

### Factors contributing to survival benefits in patients treated with sorafenib+TACE

The mean duration of sorafenib treatment was 10.8±10.51 months (median 6.1(2.5-15.74), range 1- 46.37 months. For patients treated with TACE+sorafenib, tumor size, PVTT, extrahepatic metastasis and duration of sorafenib treatment were significantly associated with survival in univariate analyses. After adjusting for tumor size, PVTT, and extrahepatic metastasis, the hazard of death decreased with increased duration of sorafenib treatment (HR=0.9, *p*<0.001). After adjusting for PVTT, extrahepatic metastasis, and duration of treatment, patients with tumor size ≥5 cm had significantly higher hazard of death compared with those with <5 cm (HR=1.54, *p*=0.01; Table 5).

**Table 5:** Factors associated with survival in unresectable HCC patients treated with TACE+sorafenib

	Univariate		Multivariate	
	HR (95% CI)	*p*-value	HR (95% CI)	*p*-value
Tumor size (cm)				
<5	Ref		Ref	
≥5	2.09(1.52-2.85)	<0.001	1.54(1.11-2.14)	0.01
PVTT				
No	Ref		Ref	
Yes	1.78(1.29-2.47)	0.001	1.1(0.78-1.56)	0.583
Extrahepatic metastasis				
No	Ref		Ref	
Yes	1.54(1.05-2.27)	0.027	1.08(0.72-1.61)	0.707
Duration of sorafenib treatment	0.89(0.87-0.91)	<0.001	0.9(0.87-0.92)	<0.001

PVTT, portal vein tumor thrombus

No significant affect in survival were found in age, gender, α-fetoprotein, Child-Pugh, tumor number, and Eastern Cooperative Oncology Group status.

Univariate analysis also revealed that tumor size, PVTT, extrahepatic metastasis and duration of sorafenib treatment were significantly associated with survival of patients with recurrence treated with TACE+sorafenib. After adjusting for tumor size, PVTT, and extrahepatic metastasis, the hazard of death decreased with increasing duration of sorafenib treatment (HR=0.9, *p*<0.001). After adjusting for PVTT, extrahepatic metastasis, and duration of sorafenib treatment, patients with tumors ≥5 cm had significantly higher hazard of death compared with those with tumors <5 cm (HR=1.83, *p*=0.031; Table 6).

**Table 6:** Factors associated with survival in uHCC patients with disease recurrence treated with TACE+sorafenib

	Univariate		Multivariate	
	HR (95% CI)	*p*-value	HR (95% CI)	*p*-value
Tumor size (cm)				
<5	Ref		Ref	
≥5	1.78(1.05-3)	0.031	1.83(1.06-3.17)	0.031
PVTT				
No	Ref		Ref	
Yes	2.25(1.36-3.73)	0.002	1.31(0.63-2.72)	0.472
Extrahepatic metastasis				
No	Ref		Ref	
Yes	2.09(1.22-3.58)	0.008	0.94(0.43-2.05)	0.881
Duration of sorafenib treatment	0.9(0.87-0.93)	<0.001	0.9(0.87-0.93)	<0.001

PVTT, portal vein tumor thrombus

No significant affect in survival were found in age, gender, α-fetoprotein, Child-Pugh, tumor number, and Eastern Cooperative Oncology Group status.

## DISCUSSION

We hypothesized that the addition of sorafenib may improve the effect of TACE in patients with recurrent HCC and that longer duration of sorafenib therapy may provide survival benefits. The addition of sorafenib to TACE provided survival benefit, particularly to patients with disease recurrence. Furthermore, multivariate analysis showed decreased hazard of death with longer duration of sorafenib treatment. In addition to treatment with sorafenib plus TACE, the survival of patients with unresectable HCC was negatively associated with the presence of PVTT. In patients with recurrence, survival was reduced by the presence of extrahepatic metastasis.

Several recent systemic reviews and meta-analyses have suggested that sorafenib with TACE may improve time to progression [24] and possibly provide survival benefits [25] in patients with unresectable HCC. In addition, the safety of this combination therapy has been shown in patients with advanced HCC [26–28]. The increased survival time observed with TACE+sorafenib in the present study is similar to that reported in a propensity score matching study comparing sorafenib plus TACE with TACE alone [29]. The overall survival of patients with advanced HCC without portal vein invasion was significantly increased in the combination therapy group compared with the monotherapy group (7.0 vs. 4.9 months, respectively) [29]. In patients with Child-Pugh class A and Barcelona Clinic Liver Cancer stage B (BCLC-B) HCC, sorafenib plus TACE increased the median time to progression from 9.2 months as compared to the 4.9 months observed in patients treated with TACE plus placebo [30]. In contrast, no differences in TTP were observed in the global phase II randomized, double blind, placebo-controlled SPACE trial (Sorafenib or placebo in combination with TACE with DEBDOX) that included 307 patients with intermediate-stage HCC treated with sorafenib plus TACE with doxorubicin-eluting beads (DEB) or placebo plus DEB-TACE (median TTP of 169 vs. 166 days) [31].

In the present study where most patients underwent sorafenib within 60 days after the first application of TACE and some even received sorafenib prior to TACE, the median survival time was 20.23 and 13.97 months in patients with unresectable HCC treated with TACE+sorafenib and TACE alone, respectively. The median survival time for patients with recurrence in the TACE+sorafenib group was 30.7 months compared with 18.22 months in the TACE group. The median survival time for 62 Chinese patients with unresectable HCC treated with TACE+sorafenib in an interim subgroup analysis of the START trial was 16.5 months [32]; it was 7 months in a propensity score matching study that included 280 patients with advanced HCC [24] and 10.7 months in a GIDEON Chinese subgroup analysis [33]. It is possible that survival times may be dependent upon the point at which sorafenib was initiated. In the present study, 27.2% of patients received sorafenib prior to TACE, and the remaining patients received it within 1 month following TACE. A greater proportion of patients in the present study had more favorable AFP, and fewer had PVTT. Thus, for patients with disease recurrence, the addition of sorafenib to TACE at an earlier stage (i.e., prior to progression to PVTT or worsening AFP) may confer additional survival benefit.

To identify factors associated with the improved survival with TACE+sorafenib combination therapy, we performed univariate and multivariate analyses. Patients with unresectable HCC and recurrence treated with TACE+sorafenib had a significantly lower hazard of death compared with those treated with TACE alone in both groups. This is consistent with another case-controlled study that assessed sorafenib in patients with HCC recurrence following liver transplantation [12]. Moreover, the relationship between extrahepatic metastasis and survival observed in those with recurrence is consistent with a study by Inghilesi et al. [34] in which extrahepatic spread was associated with overall survival in HHC patients. In addition, we showed decreased hazard of death with longer duration of sorafenib treatment in these patients, which is consistent with the observations from the Liver Cancer Study Group consensus workshop in which long-term sorafenib treatment was related to survival of HCC patients [20]. The mean duration of sorafenib treatment was 43 weeks (10.8 months) with a median of 25 weeks (6.1 months) in our study. In contrast, Kudo et al. [21], which did not find a benefit with TACE+sorafenib over TACE+placebo in terms of overall survival or time to progression in patients who responded to TACE, the median duration was 17 weeks. In addition, sorafenib was initiated >9 weeks after TACE in more than half of the patients, leading the authors to conclude that the absence of response to sorafenib may be a result of delays in starting sorafenib after TACE and/or low daily sorafenib doses [21]. Moreover, in Kudo et al.'s study [21], the Korean subgroup benefited more from the combination therapy compared with the Japanese subgroup, which may be, at least in part, explained by the longer duration of sorafenib treatment in the Korean group as compared to the Japanese subgroup. In addition to differences in sorafenib duration, different patient populations may explain the conflicting response to sorafenib given that Kudo et al. [21] focused on patients who responded to TACE while only 40% of the patients in our study achieved a complete response.

Differences in the clinical management of HCC exist between the Asia-Pacific area and Western countries. In China, TACE is the treatment of choice for patients without decompensated liver function, a single nodule of < 5 cm, or multifocal HCC without extrahepatic spread [3]. In contrast, American Association for the Study of Liver Diseases (AASLD) and EASL guidelines recommend treatment according to BCLC stage [35]. For example, surgery is an acceptable treatment strategy for patients with diseases beyond BCLC-A in the Asia-Pacific area, but it is not recommended in Western guidelines (e.g., AASLD and EASL). In contrast, sorafenib is recommended by the Western guidelines for patients with advanced BCLC-C stage disease, but has limited availability in many Asia-Pacific countries. However, in both regions, TACE is the standard therapy for unresectable HCC patients.

The present study is limited in its retrospective design. Moreover, the matching method applied in this study was intended for data collection and not for statistically balancing the bias between the two treatment groups; therefore, additional studies using propensity score analysis or matching according to disease stage or liver function are warranted. Also, patients were only included if they received sorafenib >1 month, and those who died from treatment-related complication were excluded. In addition, the time to progression, objective response rate, incidence of adverse events, and treatment-induced death were not determined. Furthermore, the heterogeneity of the patients, especially regarding sorafenib initiation, is a study limitation. Finally, because sorafenib is not readily available to Asian-Pacific patients due to different clinical practice pattern, it is likely that the patients in the TACE+sorafenib group paid for it themselves, creating a potential bias in that these patients were better off financially than the general population. This could also indicate that both the patients and the treating physicians were more aggressive in their selection of anti-cancer therapy.

In conclusion, TACE combined with sorafenib may provide survival benefits, particularly for those with HCC recurrence, considering their better baseline status. This data also suggest that early initiation and longer duration of sorafenib combination therapy (with TACE) is likely to provide additional survival benefits. Further clinical studies are required to confirm these results and identify the patients most likely to benefit from this therapeutic strategy.

## MATERIALS AND METHODS

### Patients

In the present nested case-controlled study, 1126 cases of unresectable HCC from the Eastern Hepatobiliary Surgery Hospital of the Second Military Medical University between July 2007 and December 2011 were analyzed. These cases included patients that were treatment naïve as well as those with recurrent disease. A 1:1 matching was performed according to sex and age. Because the majority of patients in the present study were classified as having BCLC-B HCC, patients were also matched according to lesion size and number of lesions given that these characteristics were prognostic factors following TACE in this subgroup of patients [36]. Of these patients, 217 patients underwent sorafinib + TACE therapy, 28 received sorafinib alone, and 245 underwent TACE alone. Because one of the study objectives was to assess the association of duration of sorafenib treatment with clinical outcomes, the data from those receiving sorafinib alone (i.e., early sorafenib initiation) was combined with that from patients receiving both sorafinib + TACE (n=245). In addition, patients with recurrence after surgery were also matched 1:1 by treatment in which 127 patients were treated with TACE + sorafenib and 127 received TACE alone. The clinical stage of HCC was determined according to the BCLC classification and the Tumor Node Metastasis (TNM) staging system.

The inclusion criteria were as follows: (1) a diagnosis of unresectable (moderate to advanced) HCC; (2) age >18 y; and (3) a clinical or pathological diagnosis with HCC. Primary HCC was diagnosed according to the diagnostic criteria for HCC of the AASLD. The clinical diagnosis of HCC was based on findings from two or more imaging assessments (one of them was contrast computed tomography [CT] or magnetic resonance imaging [MRI]), with or without an increase in serum tumor markers; liver function was classified Child-Pugh A or B; an ECOG performance score of 0-2; no history of radiotherapy and/or chemotherapy before recruitment; duration of sorafinib therapy for >1 month; white blood cells >2000 cells/UL and platelets >50000/UL; no heart or kidney dysfunction; and no malignancy of other organs/systems. Patients who died of treatment-related complications were excluded from this study.

The following clinical information was collected from all patients: gender, age, serum hepatitis B virus surface antigen (HBsAg), AFP, total bilirubin (TBIL), albumin, aspartate aminotransferase (AST), alanine transaminase (ALT), platelet levels, prothrombin time (PT), tumor number, maximum diameter of the tumor, hepatic cirrhosis, esophageal / fundus varices, PVTT, extrahepatic metastasis, and surgical intervention.

For a recurrent tumor after surgery, the overall survival time was the time from diagnosis of recurrence to death; the recurrence time after surgery was the time from surgery to recurrence. For a recurrent tumor after treatment other than surgery, the overall survival time was the time from diagnosis of recurrence to death; the recurrence time was the time from first treatment record to recurrence.

Given the retrospective study design, the requirement to obtain informed consent was waived. This study protocol conforms to the ethical guidelines of the 1975 Declaration of Helsinki as reflected in a priori approval by the Eastern Hepatobiliary Surgery Hospital of the Second Military Medical University's human research committee.

### Procedures used in the TACE group

The Seldinger technique was used for the application of TACE. In brief, following local anesthesia, the femoral artery was punctured, and a 5-F catheter sheath was inserted, with the help of a guidewire, forward to the right or left hepatic artery via the abdominal aorta / celiac / hepatic artery by digital subtraction angiography (DSA). The following chemotherapeutics were applied in this procedure: 5-FU (250-1000 mg/m^2^), hydroxycamptothecin (10-15 mg), Pirarubicin (20 mg), cisplatin (40 mg/m^2^), Mitomycin C (MMC, 10 mg/m^2^) and iodipin (2-8 mL). DSA was performed first, and chemotherapy was applied by perfusion with 5-FU and cisplatin. Subsequently, MMC, epirubicin and super-liquefacted iodipin were injected via the catheter. On the basis of tumor size and blood flow at the artery, the maximum volume was 20 mL at single injection. Finally, the vessels were embolized with a gelatin sponge containing 40 mg of gentamicin. At 1 month after surgery, an abdominal-enhanced CT was performed to evaluate tumor size, and serum AFP levels were measured to determine if a subsequent application of TACE was necessary. Generally, TACE was done once every 2-3 months. TACE was discontinued in the presence of liver function deterioration, severe complications or disease progression.

### Procedures used for sorafinib therapy in combination with TACE

Oral sorafenib was administered before (n=59) or after TACE (n=158); 97 patients received sorafenib therapy within 1 month after TACE, and 61 received sorafenib therapy at 1 month following TACE. TACE was performed as described above, and sorafenib (400 mg) was continuously administered twice daily during the therapy period. In the event of intolerable toxic reactions, sorafenib therapy lasting for more than 30 days or disease progression following sorafenib therapy, sorafenib therapy was discontinued.

### Follow-up

All of the patients underwent routine monthly follow-up at which time the following assessments were determined: abdominal ultrasonography, liver function, routine blood test, and serum AFP measurement. Abdominal-enhanced CT, MRI or DSA was performed once every 3 months. The last follow-up was conducted on December 1, 2011. The median duration of follow up was 35.8 months (range: 0.70-54.10 months).

### Statistical analysis

Analysis started from the date of diagnosis. Continuous data were grouped into categorical data; therefore, chi-square tests were performed for comparing the differences between subjects treated with TACE+sorafenib and TACE alone. Kaplan-Meier curves with log-rank tests were used to compare the survival rates of subjects treated in TACE+sorafenib and TACE groups. Univariate and multivariate Cox proportional hazard models were used to identify the factors associated with patient survival. Factors which were significantly associated with survival in the univariate model were included in the multivariate model. Statistical analyses were performed using IBM SPSS statistical software version 22 for Windows (IBM, Armond, NY, USA). A two-tailed *p*-value <0.05 indicated statistical significance.

## SUPPLEMENTARY FIGURES


