# Supplementary material for: Retrospective analysis of transarterial chemoembolization and sorafenib in Chinese patients with unresectable and recurrent hepatocellular carcinoma
Source: Oncotarget. 2016 Aug 23;7(50):83806–16. doi: 10.18632/oncotarget.11514 (PMC5347807; doi:10.18632/oncotarget.11514)
Supplement: Supplementary file 1 [file oncotarget-07-83806-s001.pdf]

Retrospective analysis of transarterial chemoembolization and sorafenib in Chinese patients with unresectable and recurrent hepatocellular carcinoma

SUPPLEMENTARY FIGURES

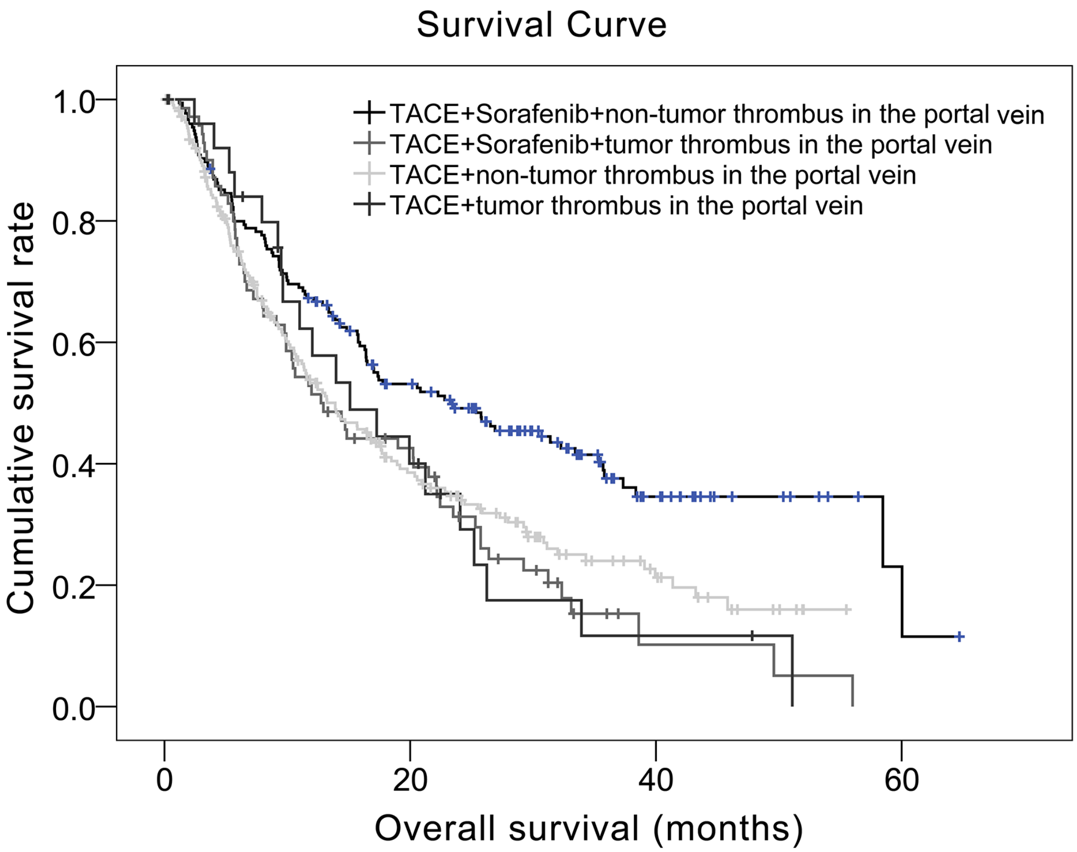

Supplementary Figure S1: Survival curves for patients with unresectable HCC treated with TACE+sorafenib or TACE alone combined with PVTT.

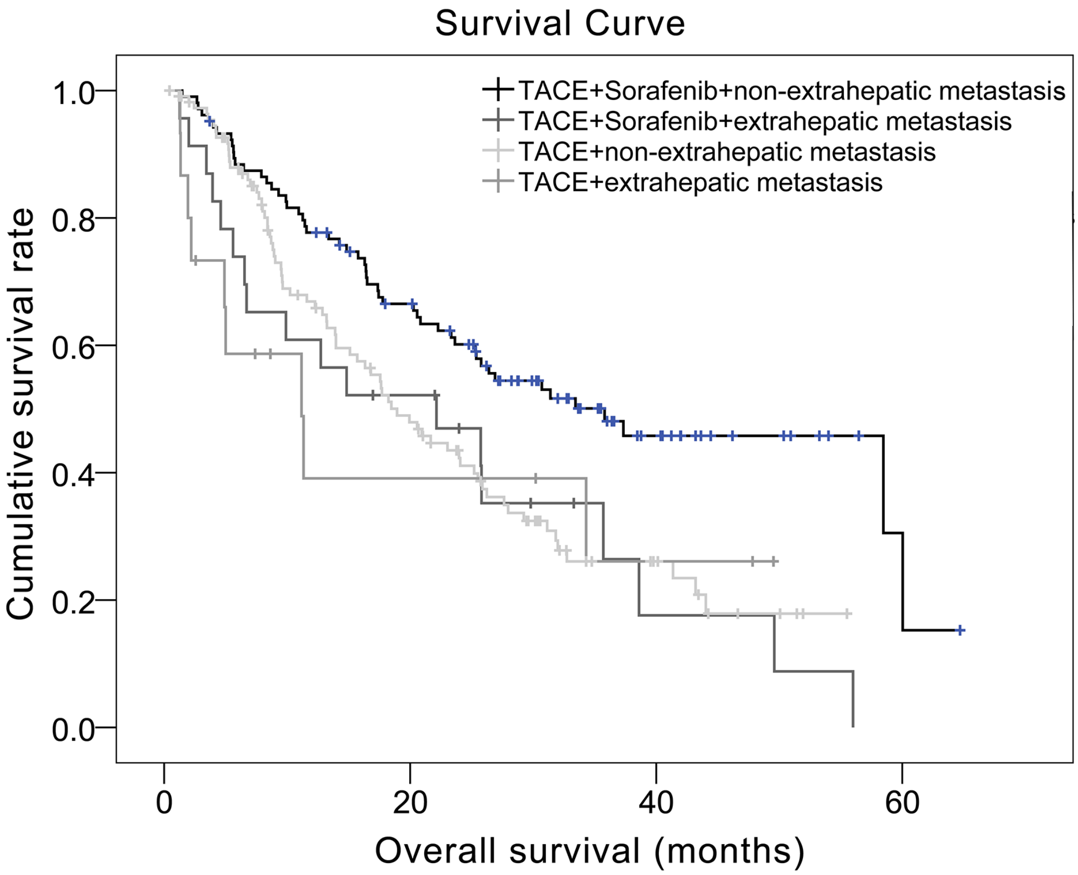

Supplementary Figure S2: Survival curves for advanced HCC patients treated with TACE+sorafenib or TACE alone combined with extrahepatic metastasis with disease recurrence.
